# 2-Oxoindolin-3-yl acetate

**DOI:** 10.1107/S1600536811009093

**Published:** 2011-03-15

**Authors:** Qiang Deng

**Affiliations:** a Xi’an Shiyou University, College of Chemistry and Chemical Engineering, Second Dianzi Road No.18, Xi’an 710065, Xi’an, People’s Republic of China

## Abstract

In the title compound, C_10_H_9_NO_3_, the mean plane through the acetate group forms a dihedral angle of 83.39 (5)° with the plane of the indole ring system. In the crystal, pairs of centrosymmetrically related mol­ecules are linked into dimers by N—H⋯O hydrogen bonds. The dimers are further connected into layers parallel to the *bc* plane by C—H⋯O hydrogen bonds.

## Related literature

For the synthesis and applications of indole-2,3-dione derivatives, see: Chen, He *et al.* (2009 ▶); Chen, Wang *et al.* (2009 ▶); Chen, Hao *et al.* (2010 ▶); Chen, Tang *et al.* (2010 ▶).
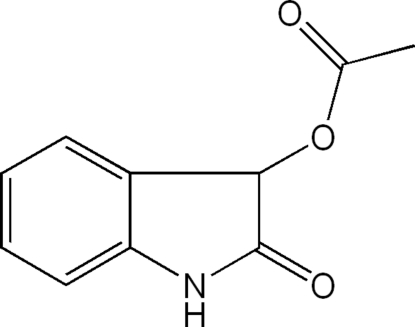


## Experimental

### 

#### Crystal data

C_10_H_9_NO_3_
*M* *_r_* = 191.18
Monoclinic, 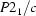

*a* = 10.617 (2) Å
*b* = 12.256 (2) Å
*c* = 7.4453 (14) Å
β = 106.347 (2)°
*V* = 929.6 (3) Å^3^
*Z* = 4
Mo *K*α radiation
μ = 0.10 mm^−1^
*T* = 296 K
0.35 × 0.30 × 0.30 mm

#### Data collection

Bruker SMART APEX CCD diffractometer
Absorption correction: multi-scan (*SADABS*; Bruker, 2002 ▶) *T*_min_ = 0.977, *T*_max_ = 0.989
4286 measured reflections
1648 independent reflections
1348 reflections with *I* > 2σ(*I*)
*R*_int_ = 0.018

#### Refinement

*R*[*F* ^2^ > 2σ(*F* ^2^)] = 0.037
*wR*(*F* ^2^) = 0.129
*S* = 1.01
1648 reflections
127 parameters
H-atom parameters constrained
Δρ_max_ = 0.24 e Å^−3^
Δρ_min_ = −0.17 e Å^−3^

### 

Data collection: *SMART* (Bruker, 2002 ▶); cell refinement: *SAINT* (Bruker, 2002 ▶); data reduction: *SAINT*; program(s) used to solve structure: *SHELXS97* (Sheldrick, 2008 ▶); program(s) used to refine structure: *SHELXL97* (Sheldrick, 2008 ▶); molecular graphics: *ORTEP-3* (Farrugia, 1997 ▶); software used to prepare material for publication: *WinGX* (Farrugia, 1999 ▶).

## Supplementary Material

Crystal structure: contains datablocks I, global. DOI: 10.1107/S1600536811009093/rz2559sup1.cif

Structure factors: contains datablocks I. DOI: 10.1107/S1600536811009093/rz2559Isup2.hkl

Additional supplementary materials:  crystallographic information; 3D view; checkCIF report

## Figures and Tables

**Table 1:** Hydrogen-bond geometry (Å, °)

*D*—H⋯*A*	*D*—H	H⋯*A*	*D*⋯*A*	*D*—H⋯*A*
N1—H1*A*⋯O1^i^	0.86	2.03	2.8819 (19)	169
C2—H2*A*⋯O1^ii^	0.98	2.44	3.394 (2)	164
C4—H4*A*⋯O3^iii^	0.93	2.56	3.328 (3)	141

Symmetry codes: (i) 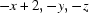; (ii) 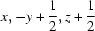; (iii) 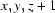.

**Fig. 1.**
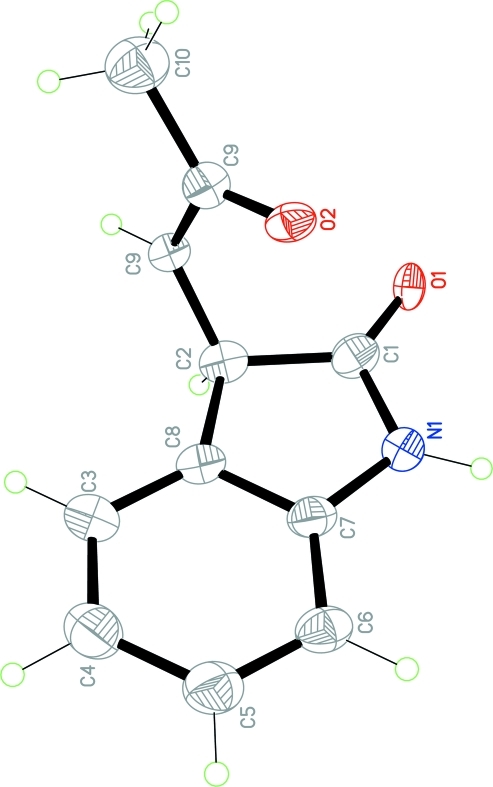
The molecular structure of the title compound, with 30% probability displacement ellipsoids.

**Fig. 2.**
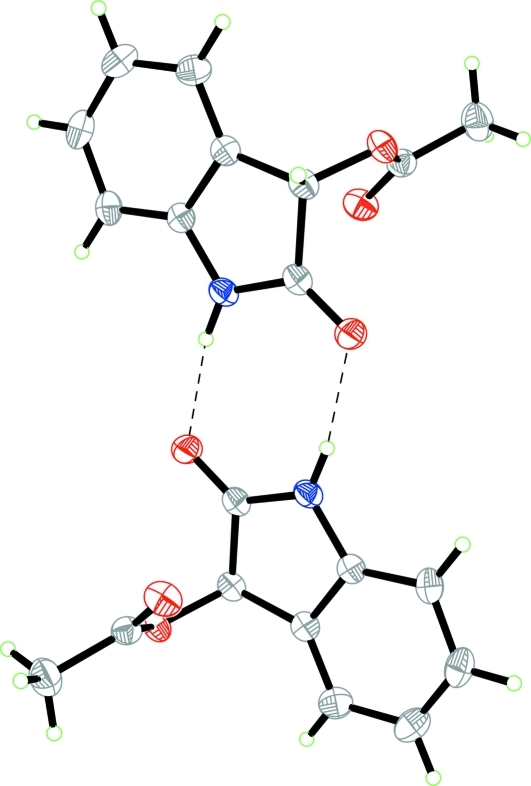
A dimer of the title compound. Intermolecular hydrogen bonds are drawn as dashed lines. Displacement ellipsoids are drawn at he 30% probability level.

## supplementary crystallographic information

### Comment

Indole-2,3-dione derivatives have driven much attention for their anti-bacterial, anti-virus and neuroprotective activities (Chen, He *et al.*, 2009; Chen, Wang *et al.*, 2009; Chen, Hao *et al.*, 2010; Chen, Tang *et al.*, 2010). The title compound, whose structure is reported herein, has been synthesized by reduction of isatin with sodium borohydride followed by acylation. The X-ray structural analysis of the title compound revealed the molecular structure as depicted in Fig. 1. Geometric parameters are in the usual ranges. In the molecule, the mean plane through the ester group (O2/O3/C9/C10) is almost perpendicular to the plane of the indole ring system, forming a dihedral angle of 83.39 (5)°. In the crystal structure, centrosymmetrically related molecules are linked into dimers by N—H···O hydrogen bonds (Fig. 2; Table 1). The dimers are further connected into a layers parallel to the *bc* plane by weak C—H···O hydrogen bonds.

### Experimental

To a solution of isatin (1.0 mmol) in methanol (20 ml), sodium borohydride (1.0 mmol) in methanol (10 ml) was added dropwise until the disappearance of isatin, as evidenced by thin-layer chromatography, then diluted hydrochloric acid (0.1 *M*) was added dropwise to eliminate the excess sodium borohydride. The solvent was removed *in vacuo*, and the residue was dissolved in 10 ml pyridine. Acetic anhydride (1.0 mmol) was then added, and the mixture was refluxed for 1 h. On completion of the reaction, the solvent was removed *in vacuo*, and the residue was separated by column chromatography (silica gel; petroleum ether/ethyl acetate 5:1 *v*/*v*), giving the title compound. 30 mg of the title compound was dissolved in 30 ml me thanol and the solution was kept at room temperature for 7 d, to give colourless single crystals suitable for X-ray analysis on slow evaporation of the solvent.

### Refinement

All H atoms were placed at calculated positions and refined as riding, with C—H = 0.93–0.98 Å, N—H = 0.86 Å, and with *U*_iso_(H) = 1.2 *U*_eq_(C, N) or 1.5 *U*_eq_(C) for methyl H atoms.

### Crystal data

**Table:**

C_10_H_9_NO_3_	*F*(000) = 400
*M**_r_* = 191.18	*D*_x_ = 1.360 Mg m^−^^3^
Monoclinic, *P*2_1_/*c*	Mo *K*α radiation, λ = 0.71073 Å
Hall symbol: -P 2ybc	Cell parameters from 7180 reflections
*a* = 10.617 (2) Å	θ = 1.6–25.0°
*b* = 12.256 (2) Å	µ = 0.10 mm^−^^1^
*c* = 7.4453 (14) Å	*T* = 296 K
β = 106.347 (2)°	Block, colourless
*V* = 929.6 (3) Å^3^	0.35 × 0.30 × 0.30 mm
*Z* = 4	

### Data collection

**Table:**

Bruker SMART APEX CCD diffractometer	1648 independent reflections
Radiation source: fine-focus sealed tube	1348 reflections with *I* > 2σ(*I*)
graphite	*R*_int_ = 0.018
φ and ω scans	θ_max_ = 25.1°, θ_min_ = 2.6°
Absorption correction: multi-scan (*SADABS*; Bruker, 2002)	*h* = −12→10
*T*_min_ = 0.977, *T*_max_ = 0.989	*k* = −14→14
4286 measured reflections	*l* = −8→8

### Refinement

**Table:**

Refinement on *F*^2^	Primary atom site location: structure-invariant direct methods
Least-squares matrix: full	Secondary atom site location: difference Fourier map
*R*[*F*^2^ > 2σ(*F*^2^)] = 0.037	Hydrogen site location: inferred from neighbouring sites
*wR*(*F*^2^) = 0.129	H-atom parameters constrained
*S* = 1.01	*w* = 1/[σ^2^(*F*_o_^2^) + (0.095*P*)^2^] where *P* = (*F*_o_^2^ + 2*F*_c_^2^)/3
1648 reflections	(Δ/σ)_max_ < 0.001
127 parameters	Δρ_max_ = 0.24 e Å^−^^3^
0 restraints	Δρ_min_ = −0.17 e Å^−^^3^

### Special details

**Table:**

Geometry. All e.s.d.'s (except the e.s.d. in the dihedral angle between two l.s. planes) are estimated using the full covariance matrix. The cell e.s.d.'s are taken into account individually in the estimation of e.s.d.'s in distances, angles and torsion angles; correlations between e.s.d.'s in cell parameters are only used when they are defined by crystal symmetry. An approximate (isotropic) treatment of cell e.s.d.'s is used for estimating e.s.d.'s involving l.s. planes.
Refinement. Refinement of *F*^2^ against ALL reflections. The weighted *R*-factor *wR* and goodness of fit *S* are based on *F*^2^, conventional *R*-factors *R* are based on *F*, with *F* set to zero for negative *F*^2^. The threshold expression of *F*^2^ > σ(*F*^2^) is used only for calculating *R*-factors(gt) *etc*. and is not relevant to the choice of reflections for refinement. *R*-factors based on *F*^2^ are statistically about twice as large as those based on *F*, and *R*- factors based on ALL data will be even larger.

### Fractional atomic coordinates and isotropic or equivalent isotropic displacement parameters (Å^2^)

**Table:**

	*x*	*y*	*z*	*U*_iso_*/*U*_eq_	
O2	0.67729 (11)	0.18607 (9)	0.11848 (15)	0.0419 (3)	
O1	0.91573 (12)	0.13689 (9)	−0.01094 (17)	0.0479 (4)	
O3	0.63567 (12)	0.03276 (10)	−0.05054 (17)	0.0509 (4)	
N1	0.92299 (13)	−0.00916 (10)	0.18474 (19)	0.0386 (4)	
H1A	0.9772	−0.0495	0.1478	0.046*	
C7	0.86451 (16)	−0.03943 (12)	0.3255 (2)	0.0351 (4)	
C2	0.79735 (15)	0.13809 (12)	0.2308 (2)	0.0373 (4)	
H2A	0.8479	0.1957	0.3107	0.045*	
C8	0.78545 (16)	0.04492 (13)	0.3545 (2)	0.0370 (4)	
C1	0.88381 (15)	0.09027 (13)	0.1153 (2)	0.0371 (4)	
C6	0.87970 (17)	−0.13532 (13)	0.4245 (2)	0.0414 (4)	
H6A	0.9337	−0.1909	0.4041	0.050*	
C9	0.60217 (16)	0.12244 (13)	−0.0200 (2)	0.0402 (4)	
C5	0.81038 (19)	−0.14553 (15)	0.5569 (2)	0.0522 (5)	
H5A	0.8167	−0.2102	0.6244	0.063*	
C3	0.72078 (19)	0.03455 (15)	0.4902 (3)	0.0494 (5)	
H3A	0.6700	0.0916	0.5141	0.059*	
C10	0.47947 (19)	0.17936 (17)	−0.1220 (3)	0.0600 (6)	
H10A	0.4289	0.1330	−0.2199	0.090*	
H10B	0.5009	0.2457	−0.1755	0.090*	
H10C	0.4293	0.1962	−0.0366	0.090*	
C4	0.7324 (2)	−0.06214 (17)	0.5909 (3)	0.0561 (5)	
H4A	0.6877	−0.0708	0.6810	0.067*	

### Atomic displacement parameters (Å^2^)

**Table:**

	*U*^11^	*U*^22^	*U*^33^	*U*^12^	*U*^13^	*U*^23^
O2	0.0401 (7)	0.0339 (6)	0.0487 (7)	0.0061 (5)	0.0075 (5)	−0.0026 (5)
O1	0.0483 (8)	0.0417 (7)	0.0596 (8)	0.0022 (5)	0.0247 (6)	0.0110 (6)
O3	0.0482 (8)	0.0489 (8)	0.0540 (8)	−0.0011 (6)	0.0120 (6)	−0.0137 (6)
N1	0.0360 (7)	0.0337 (7)	0.0471 (8)	0.0039 (6)	0.0133 (6)	−0.0002 (6)
C7	0.0332 (8)	0.0352 (9)	0.0335 (8)	−0.0036 (6)	0.0035 (7)	−0.0055 (6)
C2	0.0359 (9)	0.0322 (8)	0.0412 (9)	−0.0002 (7)	0.0067 (7)	−0.0054 (6)
C8	0.0379 (9)	0.0347 (9)	0.0358 (8)	−0.0019 (6)	0.0061 (7)	−0.0067 (6)
C1	0.0315 (8)	0.0319 (8)	0.0456 (9)	−0.0015 (6)	0.0070 (7)	−0.0017 (7)
C6	0.0448 (10)	0.0341 (9)	0.0385 (9)	−0.0010 (7)	0.0005 (7)	−0.0008 (7)
C9	0.0389 (9)	0.0408 (10)	0.0428 (9)	−0.0021 (7)	0.0148 (8)	0.0005 (7)
C5	0.0627 (12)	0.0488 (11)	0.0376 (9)	−0.0080 (9)	0.0020 (9)	0.0066 (8)
C3	0.0559 (11)	0.0500 (11)	0.0449 (10)	0.0008 (8)	0.0181 (9)	−0.0090 (8)
C10	0.0486 (11)	0.0603 (12)	0.0637 (12)	0.0017 (9)	0.0037 (10)	0.0078 (10)
C4	0.0689 (14)	0.0615 (12)	0.0418 (10)	−0.0084 (10)	0.0221 (10)	−0.0012 (8)

### Geometric parameters (Å, °)

**Table:**

O2—C9	1.3582 (19)	C8—C3	1.378 (2)
O2—C2	1.4385 (18)	C6—C5	1.392 (3)
O1—C1	1.2263 (19)	C6—H6A	0.9300
O3—C9	1.1965 (19)	C9—C10	1.484 (2)
N1—C1	1.343 (2)	C5—C4	1.383 (3)
N1—C7	1.410 (2)	C5—H5A	0.9300
N1—H1A	0.8600	C3—C4	1.389 (3)
C7—C6	1.372 (2)	C3—H3A	0.9300
C7—C8	1.386 (2)	C10—H10A	0.9600
C2—C8	1.495 (2)	C10—H10B	0.9600
C2—C1	1.539 (2)	C10—H10C	0.9600
C2—H2A	0.9800	C4—H4A	0.9300
			
C9—O2—C2	116.20 (12)	C7—C6—H6A	121.6
C1—N1—C7	111.81 (13)	C5—C6—H6A	121.6
C1—N1—H1A	124.1	O3—C9—O2	121.95 (15)
C7—N1—H1A	124.1	O3—C9—C10	126.84 (16)
C6—C7—C8	122.64 (16)	O2—C9—C10	111.21 (15)
C6—C7—N1	127.95 (15)	C4—C5—C6	121.75 (17)
C8—C7—N1	109.41 (14)	C4—C5—H5A	119.1
O2—C2—C8	117.11 (13)	C6—C5—H5A	119.1
O2—C2—C1	113.65 (12)	C8—C3—C4	119.17 (17)
C8—C2—C1	102.74 (12)	C8—C3—H3A	120.4
O2—C2—H2A	107.6	C4—C3—H3A	120.4
C8—C2—H2A	107.6	C9—C10—H10A	109.5
C1—C2—H2A	107.6	C9—C10—H10B	109.5
C3—C8—C7	119.61 (15)	H10A—C10—H10B	109.5
C3—C8—C2	131.95 (15)	C9—C10—H10C	109.5
C7—C8—C2	108.27 (14)	H10A—C10—H10C	109.5
O1—C1—N1	126.53 (15)	H10B—C10—H10C	109.5
O1—C1—C2	125.98 (15)	C5—C4—C3	119.92 (18)
N1—C1—C2	107.41 (13)	C5—C4—H4A	120.0
C7—C6—C5	116.86 (16)	C3—C4—H4A	120.0

### Hydrogen-bond geometry (Å, °)

**Table:**

*D*—H···*A*	*D*—H	H···*A*	*D*···*A*	*D*—H···*A*
N1—H1A···O1^i^	0.86	2.03	2.8819 (19)	169
C2—H2A···O1^ii^	0.98	2.44	3.394 (2)	164
C4—H4A···O3^iii^	0.93	2.56	3.328 (3)	141

Symmetry codes: (i) −*x*+2, −*y*, −*z*; (ii) *x*, −*y*+1/2, *z*+1/2; (iii) *x*, *y*, *z*+1.
